# Caudal epidural catheterization for pain management in 48 hospitalized horses: A descriptive study of demographics, complications, and outcomes

**DOI:** 10.3389/fvets.2022.995299

**Published:** 2022-10-28

**Authors:** Hope Douglas, Monica Midon, Kavita Shroff, Dario Floriano, Bernd Driessen, Klaus Hopster

**Affiliations:** Department of Clinical Studies, New Bolton Center, School of Veterinary Medicine, University of Pennsylvania, Kennett Square, PA, United States

**Keywords:** horse, epidural, analgesia, morphine, pain management

## Abstract

The placement of caudal epidural catheters in horses has become more frequent as a multi-modal analgesic strategy. Despite its integration into clinical practice, there are limited reports describing the use of caudal epidural catheterization for prolonged use in horses. The purpose of this study was to characterize the hospitalized caseload undergoing epidural catheterization for long-term epidural analgesic administration, to report the response to epidural therapy and observed complications, and to describe patient outcomes. Medical records of hospitalized equine patients that underwent placement of a caudal epidural catheter for analgesic management between 2017 and 2021 were analyzed retrospectively. For the 62 catheters placed in the 48 cases, the most frequent diagnosis category prompting epidural analgesia was orthopedic (43/48, 89.6%). Synovial sepsis was the most frequent specific diagnosis prompting epidural catheter placement (11/48, 22.9%). The initial response to epidural therapy was characterized as positive for 37/62 (59.7%) catheters. Complications were documented for 46/62 (74.2%) catheters. However, most of these complications were classified as mild (51.6%) or moderate (14.5%), and exaggerated physiologic responses were observed most frequently. Of the horses studied, 52.1% survived to be discharged from the hospital. With awareness of potential complications and vigilant monitoring, caudal epidural catheters should be considered for equine patients as an analgesic strategy.

## Introduction

Epidural analgesia is a well-documented technique that has been demonstrated to provide effective pain management in human and veterinary medicine (1–4). The high efficacy of this strategy has resulted in its perioperative use as well as its integration into analgesic protocols for hospitalized equine patients (3, 5–11). Horses, due to their unique physiologic and conformational characteristics, are at risk of adverse effects of high-dose and long-term administration of non-steroidal anti-inflammatory medications and opioids (3, 12–14). In addition, the unique issues regarding the development of laminitis have prompted the development and use of long-term locoregional and neuraxial techniques (15, 16).

The placement of epidural catheters for continuous caudal epidural therapy in horses was first reported in 1983 and has become more frequently implemented clinically to employ multi-modal analgesic therapies (3, 14, 17–24). Caudal epidural catheters have been placed for continuous anesthesia and analgesia for surgical procedures as well as for pain management for orthopedic, rectal, and urogenital conditions (3, 14, 18, 19, 21, 25). Continuous or repeated epidural administration of analgesics offers the advantages of effective analgesia with reportedly fewer side effects than systemic analgesics (26, 27). Despite its more frequent application in experimental and clinical settings, there are limited reports on the use of caudal epidural catheterization for long-term administration of epidural analgesics (18, 21, 24, 25). In an experimental, prospective study on ten horses, no catheter-related complications, such as dislocation, catheter obstruction, leakage, or discomfort during injection were observed during a 14-day experimental observation period (24). Another retrospective study of 50 epidural catheters reported 3 temporary patient-related complications and 22 technical problems associated with the epidural catheters (18). The response to epidural treatment was considered negative in only 4 horses, and based on these results, it was concluded that catheterization is an effective technique for repeated delivery of epidural analgesics in horses (18).

While we routinely integrate epidural catheterization and epidural analgesia administration into our clinical practice, it has been our clinical impression that the frequency and description of complications has been under-reported (3, 17, 18, 24, 28). Thus, the purpose of this study was to characterize the hospitalized caseload undergoing epidural catheterization for long-term epidural analgesic administration, to report the response to epidural therapy and observed complications, and to describe patient outcomes.

## Materials and methods

The medical records of equine patients hospitalized at the University of Pennsylvania's New Bolton Center George D. Widener Hospital for Large Animals from January 2017 until December 2021 that had undergone placement of a caudal epidural catheter for analgesic management were analyzed retrospectively. The hospital's electronic medical record system was queried using the charge codes for epidural catheter placement and for the epidural catheter kits. From this list of records, cases were selected that had an epidural catheter placed for long-term (>24 h) analgesic therapy administration during hospitalization.

### Case information

Electronic and handwritten medical records for each case were scrutinized individually. Signalment information (age, breed, sex, body weight) was recorded for each horse. The diagnosis prompting epidural analgesia was categorized broadly as orthopedic, abdominal, reproductive, or other. This diagnosis was further categorized as either synovial sepsis (septic arthritis, tenosynovitis, bursitis), cellulitis, surgical site infection, limb fracture, pelvic fracture, trauma, or other. The reason for epidural analgesia was recorded for each catheter.

### Catheter information

**Figure 1:**
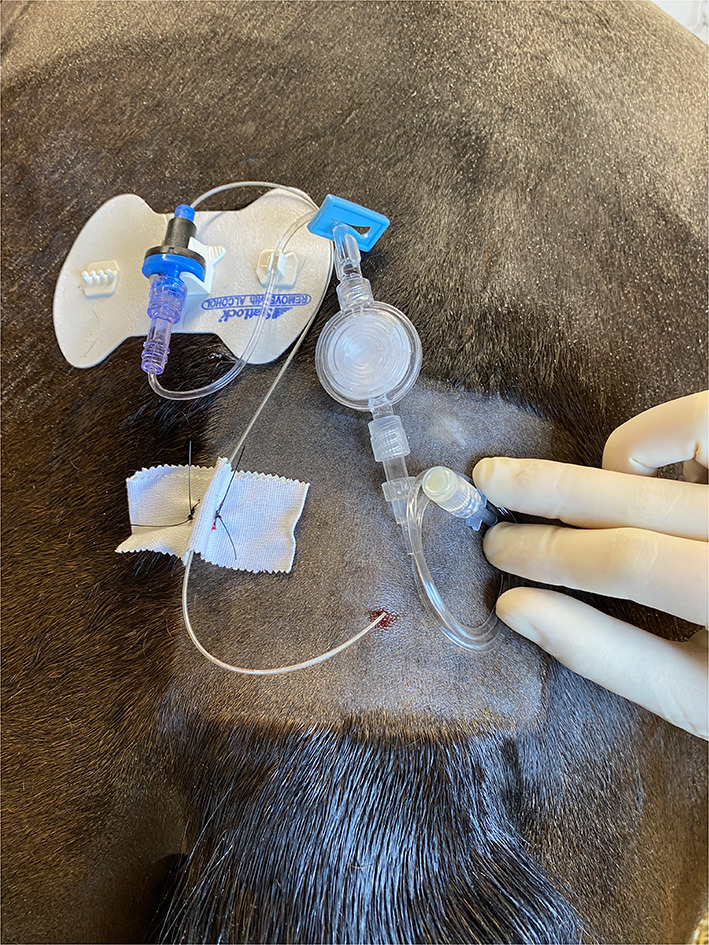
Caudal epidural catheter secured to the skin.

Each new or replacement epidural catheter was considered uniquely for epidural catheter analysis. In all cases the Arrow^®^ FlexBlock^TM^ Continuous Peripheral Nerve Block Kits (Teleflex^®^, Reading, PA, USA) were used. These kits contain a 19 Ga × 60 cm continuous nerve block catheter, and they have been used clinically for long-term epidural analgesia in horses. After aseptic preparation of the sacrococcygeal or first intercoccygeal space, 2% lidocaine was used for local anesthesia of the skin. After confirmation of placement of the 17 Ga × 3” (8 cm) Tuohy needle into the epidural space using the Hanging Drop Technique and Loss of Resistance Technique, the catheter was advanced to a distance appropriate for the specific condition (3, 19). For hindlimb orthopedic conditions, a distance of 25 cm was targeted. For abdominal, thoracic, or forelimb conditions, the catheter was advanced as far as possible. After placement of the epidural catheter, the SnapLock^TM^ catheter syringe adaptor was secured to the catheter. The provided 6” extension tubing was attached to the syringe adaptor, and the 0.2 micron flat filter was added. The provided 18” extension tubing was then attached to the filter, and an injection cap was added to the end of the system. The filter and the extension sets were flushed with saline prior to system attachment. Using the provided STATLOCK^®^ stabilization device, the catheter system was adhered to the patient. Based on individual clinician preference, cyanoacrylate glue (Super Jet^TM^, Jet Glues, Inc, Deerfield, IL, USA) was placed on the adhesive side of the stabilization device for additional security. The epidural catheter was also secured to the skin using 2-0 nylon suture (Ethilon^®^, Ethicon^TM^, Bridgewater, NJ, USA) (Figure 1), and a dressing was placed over the site for protection. The dressing typically included sterile gauze covered with adhesive elastic tape (Elastikon^®^, Johnson & Johnson, New Brunswick, NJ, USA). A multipurpose spray adhesive (Super 77^TM^, 3M^TM^, St. Paul, MN, USA) was frequently applied for additional dressing reinforcement. The exact composition and layers of the dressing and any additional adhesives used were based on clinician preference and case requirements. The duration of maintenance of each epidural catheter was determined from the time of placement to the time of removal.

### Epidural dose information and response

The epidural doses were prepared aseptically under a laminar airflow hood. The total number of dosages administered as well as the volume (total and mL/kg) of the epidural dose were recorded for each individual epidural catheter. Additionally, the composition of the epidural treatment was analyzed. The initial dose of morphine (total and mg/kg) and frequency of dosing (once, every 8 h, every 12 h, every 24 h) was identified. Dose escalation (yes/no) or dose tapering (yes/no) were defined as increased or reduced morphine dose and/or frequency during epidural analgesic treatment, respectively. The characteristics of additional agents added to the epidural doses were also recorded. If an alpha-2 agonist agent was added to an epidural dose at any point during treatment, it was categorized as yes/no. Based on individual clinician preference, nothing, a variable volume of 0.9% saline, or heparinized saline solution was “flushed” into the epidural catheter following analgesic dose administration.

The initial response to epidural therapy recorded in the patient medical record was characterized as positive, negative, mixed, or inconclusive for each individual epidural catheter using an adaptation of the scale by Martin et al. (18). The reported clinical interpretation was considered positive if improvement in patient condition was observed, negative if the patient condition did not change or worsened and/or new problems developed, mixed if the response was both positive and negative, and inconclusive if no comment was recorded (18). The response information was obtained directly from the medical record text describing the patient's clinical assessment by the attending veterinary team after the first epidural treatment.

### Epidural complications

An all-inclusive definition of a complication was adopted and was defined any negative event reported in the medical record text related to or potentially related to the epidural catheter system or epidural analgesic administration that required monitoring, intervention, or resulted in actual or potential patient harm or injury. A complication due to the epidural catheter was described as “yes” if it was documented in the medical record or “no” if was not reported in the text of the medical record. The number of complications were identified (single or multiple). Complications were categorized as none, mild (no intervention required), moderate (intervention required), or severe (significant patient injury or death) based on a modification of the scale by Dindo et al. (29). Each epidural complication was classified based on etiology using a modified scale by Martin et al. (18) and was assigned by the authors as a technical complication, a patient- related complication, and an exaggerated physiologic response. A technical complication (yes/no) was defined as a problem with the epidural catheter itself. A patient-related complication was defined as an event during which the patient caused damage to the catheter (yes/no). If a technical complication or patient-related complication occurred, the description of the problem recorded in the patient medical record was included for identification. An exaggerated physiologic response was defined as an observed or potential behavior or physiologic consequence of epidural analgesic administration based on clinical patient assessment documented in the medical record. If an exaggerated physiologic response was observed at any point during epidural treatment regardless of the number of times, it was considered as one occurrence per each individual epidural catheter (yes/no). The number of instances of each observed exaggerated physiologic response was not documented. These exaggerated physiologic responses were further described based on the text of the hospital medical record system as agitation [pacing, sweating, pawing, restlessness, perceived anxiety, increased locomotor activity (yes/no)], pruritus [scratching tail base (yes/no)], ataxia [loss of balance, falling, recumbency during or immediately after epidural injection (yes/no)], colic [signs of abdominal discomfort, pawing, flank-watching, rolling (yes/no)], urinary retention [reduced urine output (yes/no)], reduced manure production (yes/no), and other (yes/no). Reduce urine output was characterized by observed decreased frequency of urination, reduced soiling of stall bedding, or increased bladder size based on abdominal palpation per rectum or ultrasonographic assessment. Using the hospital's standard monitoring sheet, the number of manure piles recorded in the 24 h prior to, and post, epidural catheter placement was documented. Clinical interventions to treat these exaggerated physiologic responses were not standardized and were based on individual clinician preference and case considerations. Enteral fluid administration during epidural analgesic treatment was defined as the administration of any type of fluid *via* a nasogastric tube (yes/no). Feed restriction at any point during the time of epidural therapy was also recorded (yes/no).

### Hospitalization progression

Concurrent treatments during the time of epidural analgesia included systemic antibiotic administration (yes/no), regional limb perfusion or intra-articular medication administration (yes/no), general anesthesia with surgery prior to epidural catheter placement (yes/no), and general anesthesia and surgery following epidural catheter placement (yes/no). Administration of additional systemic medications was documented and included non-steroidal anti-inflammatory drug administration (phenylbutazone or flunixin meglumine), acetaminophen (yes/no), gabapentin (yes/no), trazodone (yes/no), clopidogrel (yes/no), and lidocaine constant rate infusion (yes/no). The performance of a locoregional block during epidural treatment was recorded (yes/no). Maintenance in a sling for patient management was also documented (yes/no). Signs of Systemic Inflammatory Response Syndrome (SIRS) where defined as the presence of two or more of the following: an abnormal leukocyte distribution or number, pyrexia, hypothermia, tachycardia, tachypnea (30). Signs of SIRS were identified at the time of catheter placement (yes/no) or during epidural analgesia treatment (yes/no). Any complication that occurred during hospitalization that was not attributable to the epidural catheter was recorded (yes/no). A complication was defined as any negative event that occurred during hospitalization as a consequence of treatment or intervention or the development of a new disease process (29). Complications not related to the epidural catheter or to epidural analgesia were further characterized as none, mild (no intervention required), moderate (intervention required), or severe (significant patient harm or death) (29).

### Case outcome

Case outcome was analyzed for each horse. For cases with multiple epidural catheters, case outcome analysis was performed using the data from the epidural catheter that was maintained for the longest amount of time. Discharge from the hospital was noted (yes/no), and the reason of death or euthanasia in the hospital was reported. The development of complications following discharge was also determined for each case when applicable. If a mare was pregnant at the time of epidural catheterization and treatment, the outcome of the pregnancy was determined.

### Statistical analyses

The data analysis was performed using JMP^®^16.2.0 (SAS Institute, Cary, North Carolina). Normality testing was performed to determine the skewness of the continuous data using the Shapiro-Wilk and Anderson-Darling Tests. Non-parametric continuous data were reported as median and minimum and maximum values. If the data were normally distributed, the data was described as mean ± standard deviation. Categorical variables were recorded as frequencies and percentages. The Wilcoxon Signed Rank Test for matched pairs was used to analyze the recorded manure production in the 24-h time interval pre- vs. post- epidural catheter placement. *P* < 0.05 was used as the criterion for significance.

## Results

### Case information

From January 2017 until December 2021, 48 horses underwent caudal epidural catheter placement for long-term neuraxial analgesia. One horse underwent caudal epidural catheter placement during two different hospitalizations. The median age at admission was 7.5 years (minimum 14 days—maximum 25 years). The most frequent breed was Thoroughbred/Thoroughbred-cross (23/48, 47.9%), followed by Standardbred (10/48, 20.8%), Warmblood/ Warmblood-cross (5/48, 10.4%), American breed (Quarter Horse or Paint, 4/48, 8.3%), and other (6/48, 12.5%). Epidural catheters were most frequently placed in female horses (27/48, 56.2%), followed by castrated males (15/48, 31.2%), and intact males (6/48, 12.5%). Median body weight was 500 kg (range 74.5–670 kg). The most frequent diagnosis category prompting epidural analgesia was orthopedic (43/48, 89.6%). Synovial sepsis was the most frequent specific diagnosis prompting epidural catheter placement (11/48, 22.9%) followed by other (10/48, 20.8%), cellulitis (8/48, 16.7%), limb fracture (8/48, 16.7%), pelvic fracture (5/48, 10.4%), surgical site infection (4/48, 8.3%), and trauma (2/48, 4.2%). Most catheters (34/62, 54.8%) were placed due to declining patient condition and persistent pain despite conventional systemic analgesic strategies, with 5 catheters (5/34, 14.7%) placed as replacements for non-functional epidural catheter systems.

### Catheter information

A total of 62 caudal epidural catheters were placed in 48 individual horses. Eight horses had multiple (>1) epidural catheters placed during hospitalization. Catheters were maintained for a median duration of 4 days (minimum 1 h—maximum 33 days).

### Epidural dose information and response

A median of 7 epidural doses (1–75 doses) were administered. The median volume of the initial dose of epidural analgesic was 0.07 mL/kg (0.03–0.24 mL/kg). Morphine was used in every epidural treatment, and it was diluted with 0.9% saline (Hospira, Inc., Lake Forest, IL, USA) to reach the desired total volume.

Morphine (Hospira, Inc., Lake Forest, IL, USA) was administered as either 10 mg/mL or 50 mg/mL depending on drug availability. The preservative-free formulation was selected whenever possible based on product availability. The specific details regarding the specific concentration and type of morphine used for each dose administered was not available based on the information provided by the medical record and hospital information system. The initial dose of morphine was 0.05–0.3 mg/kg of morphine (median 0.18 mg/kg). The doses were either administered initially every 8 h (38/62, 61.3%), every 12 h (16/62, 25.8%) or every 24 h (2/62, 3.2%) and once in 6 out of 62 cases (9.7%). Dose escalation was required for 6/62 (9.7%) and was not required for 52/62 (83.9%). Dose tapering was performed for 27/62 (43.5%).

An alpha-2 agonist was added to an epidural dose at any point during treatment for 21/62 (33.9%) catheter treatment protocols. Xylazine [0.13 mg/kg ± 0.04 (VETone^®^AnaSed^®^ LA, MWI, Boise, ID, USA)] was administered for all 21 of these catheters. Detomidine [0.009 mg/kg (Dormosedan^®^, Zoetis, Kalamazoo, MI, USA)] was substituted for xylazine in the epidural dose for one treatment for one horse, and dexmedetomidine [0.001–0.002 mg/kg (Dexdomitor^®^, Zoetis, Kalamazoo, MI, USA)] was added instead of xylazine for one horse for 1 day of treatment.

A local anesthetic was added to an epidural dose in 4/62 (6.4%) catheters. Local anesthetic solutions used included 2% lidocaine [*n* = 1, 0.12 mg/kg (Hospira, Inc., Lake Forest, IL, USA)], 0.2% ropivacaine [*n* = 2, 0.06–0.16 mg/kg (Hospira, Inc., Lake Forest, IL, USA)], or 0.5% bupivacaine [*n* = 1, 0.09–0.17 mg/kg (Hospira, Inc., Lake Forest, IL, USA)]. Ketamine [0.1 mg/kg (Ketathesia^TM^, Henry Schein^®^ Animal Health, Dublin, OH, USA)] was included in an epidural treatment for one horse. The epidural catheter was flushed at least once with physiologic or heparinized saline for 22/62 (35.5%) of the catheters.

The initial response to epidural therapy was characterized as positive for 37/62 catheters (59.7%), inconclusive for 11/62 (17.7%), and mixed for 10/62 (16.1%). No comment regarding the initial patient response was recorded for 4/62 (6.4%).

### Epidural complications

Complications (any number) were observed and reported for 46/62 (74.2%) of the epidural catheters, with multiple complications reported for 26 of these. Complications were considered mild for 32/62 (51.6%) of the epidural catheters, moderate for 9/62 (14.5%), and severe for 5/62 (8.1%).

Epidural complications were analyzed based on etiology. Technical complications due to problems with the epidural catheter itself were reported for 12/62 (19.3%) catheters (Table 1). Patient-related complications, involving patient-induced damage to the catheter, were reported for 14/62 (22.5%) catheters (Table 1). None of the technical or patient-related complications were considered severe. Exaggerated physiologic responses were observed most frequently of the three complication categories, and one or multiple responses were observed for 37/62 (59.7%) catheters (Table 2). Multiple types of exaggerated physiologic responses were observed for 25/37 (67.6%) instances. Agitation was the most frequently observed exaggerated physiologic response (19/62, 30.6%), followed by pruritus (18/62, 29.0%), and reduced manure production (17/62, 27.4%). Reduced manure production resulted in manual evacuation of the rectum for 5/17 (29.4%) occurrences, and a rectal tear occurred in one occurrence. Concurrent gastrointestinal distention with reduced manure production occurred in 3/17 (17.6%), reduced appetite was documented for 4/17 (23.5%), and reduced borborygmi were auscultated concurrently in 2/17 (11.7%). Signs of colic were observed during treatment for 9/62 (14.5%) epidural catheters. Colic signs were attributed to gastrointestinal stasis (*n* = 4), gas distention (*n* = 1), gastric impaction and rupture (*n* = 1), right dorsal displacement of the large colon and small intestinal volvulus leading to exploratory laparotomy (*n* = 1), large colon impaction and cecal rupture (*n* = 1), and rectal impaction (*n* = 1). Pawing was observed in occurrences of agitation (*n* = 4) and reduced manure production (*n* = 1). Ataxia caused recumbency in 6/8 (75%) occurrences. All recumbency events occurred during or immediately following dose administration, and a local anesthetic solution was not included in any of these doses (Table 3). Urinary catheterization was required for 2/3 (66.7%) occurrences of urinary retention. Skin irritation was observed infrequently and was believed to be an adverse reaction to the cyanoacrylate glue used to secure the catheter system stabilization device or to the adhesive spray or due to self-trauma from pruritic behaviors (1/62, 1.6%). Information regarding the number of piles of manure recorded in the medical record for both the 24 h prior to catheter placement and 24 h following catheter placement was available for 47 catheters. The number of piles of manure reported in the medical record in the 24-h time interval post-epidural catheter placement (median 2 piles, minimum 0—maximum 5 piles) was reduced relative to the number of piles written in the medical record in the 24-h interval prior to epidural catheter placement (median 3 piles, minimum 0—maximum 9 piles) (*P* < 0.001). Enteral fluid therapy administered *via* nasogastric tube was elected during the treatment course for 19/62 (30.6%) of the catheters. Feed was withheld therapeutically during the time of epidural treatment for 5/62 (8.1%) instances.

**Table 1:** Technical and patient-related complications observed in 62 epidural catheters (*n*, percentage).

**Technical complications (12/62, 19.3%)**	**Patient-related complications (14/62, 22.5%)**
Catheter not patent (7/12, 58.3%)	Catheter dislodged or pulled out by patient (partially or completely) (7/14, 50.0%)
Leaking from catheter (2/12, 16.7%)	Damage to protective bandage/dressing (3/14, 21.4%)
Problem with filter (2/12, 16.7%)	Damage to skin sutures (1/14, 7.1%)
Disconnection between catheter and extension tubing (1/12, 8.3%)	Inability to advance catheter to desired distance (1/14, 7.1%)
	Broken extension set (1/14, 7.1%)
	Contaminated filter (1/14, 7.1%)

**Table 2:** Exaggerated physiologic responses observed in 62 epidural catheters.

**Exaggerated physiologic response**	**Occurrences (*n*, percentage)**
Agitation	19, 30.6%
Pruritus	18, 29.0%
Reduced manure production	17, 27.4%
Colic	9, 14.5%
Ataxia	8, 12.9%
Urinary retention	3, 4.8%
Other	3, 4.8%
-Skin irritation	−1, 1.6%
- Reduced anal tone	−1, 1.6%
- Tail twitching	−1, 1.6%

**Table 3:** Occurrences of recumbency during or following epidural administration.

**Event**	**Drug**	**Dose of morphine**	**Injectate volume**	**Timing of recumbency**	**Description**
1	Morphine	0.20 mg/kg	0.2 mL/kg	During administration	Fell and got up during administration of first epidural dose
2	Morphine	0.18 mg/kg	0.22 mL/kg	Immediately post-administration	Patient uncomfortable after first epidural, fell into lateral then stood
3	Morphine	0.27 mg/kg	0.09 mL/kg	Immediately post-administration	Lifted head and fell down then righted herself and stood
4	Morphine	0.18 mg/kg	0.15 mL/kg	During administration	During administration, became acutely ataxic and fell down, stayed down 5 min then stood
5	Morphine	0.15 mg/kg	0.2 mL/kg	Immediately post-administration	After last dose of epidural, patient urinated, then stepped forward unsteadily and fell into the corner of the stall
6	Morphine	0.09 mg/kg	0.19 mL/kg	During administration	During administration, horse fell down then stood

### Hospitalization progression

Concurrent therapeutics and procedures that were administered or performed during the time of epidural analgesia are summarized in Table 4. Systemic antimicrobials were administered concurrently for 55/62 (88.7%). Clopidogrel was administered concurrently during epidural treatment for 5/62 (8.1%) catheters. A sling was used for support in 12/62 (19.3%) instances. Evidence of SIRS was present at the time of epidural catheter placement for 11/62 (17.7%), and signs of SIRS were observed during the course of epidural analgesia treatment for 18/62 (29.0%). Clinical evidence of localized infection at the site were not observed for any of the epidural catheters. None of the catheters underwent microbial analysis upon removal. Additional complications not related to the epidural catheter were observed during hospitalization in 43/62 (69.3%) instances. These complications were classified as severe (31/62, 50.0%), moderate (7/62, 11.3%), mild (5/62, 8.1%), or none (19/62, 30.6%).

**Table 4:** Concurrent treatments during caudal epidural analgesia for 62 epidural catheters.

**Treatment**	**Frequency (*n*, %)**
NSAID	62/62, 100%
- Phenylbutazone	-48/62, 77.4%
- Flunixin meglumine	-11/62, 17.7%
- Phenylbutazone and flunixin meglumine	-1/62, 1.6%
-Firocoxib	-1/62, 1.6%
- None	-1/62, 1.6%
Gabapentin	13/62, 21.0%
Trazodone	4/62, 6.4%
Acetaminophen	4/62, 6.4%
Lidocaine constant rate infusion	10/62, 16.1%
Local block	14/62, 22.6%
Regional limb perfusion/intra-articular medication	27/62, 43.5%
General anesthesia/surgery prior to epidural catheter	24/62, 38.7%
General anesthesia/surgery post epidural catheter	18/62, 29.0%

### Case outcome

Survival to discharge from the hospital was achieved for 25/48 horses (52.1%). Reasons for death or euthanasia for the 23 non-survivors are described in Table 5. The contribution of, or the possibility of a contribution of, the epidural analgesia to death or euthanasia cannot be excluded for 4 horses: 2 with gastrointestinal rupture (1 gastric and 1 cecal), 1 with a transverse metacarpal fracture that occurred associated with a transfixation pin hole in a horse upon standing after falling during epidural dose administration, and 1 with a severe pelvic fracture with secondary hemoabdomen that died in the hours following epidural catheter placement and dose administration. Five horses (5/48, 10.4%) had known complications after discharge reported during the study period. None of these complications were related to the epidural catheter site. Six (6/48, 12.5%) horses were pregnant at the time of epidural catheter placement and treatment. One mare delivered a live foal in hospital after epidural treatments had ceased. One mare aborted a foal during epidural treatment prior to euthanasia due to large colon impaction and cecal rupture. One mare had known *in utero* fetal death during hospitalization and epidural treatment, and an *in utero* fetal death occurred during hospitalization for a second mare after epidural treatments had ceased. Although two mares were still pregnant at the time of discharge, no foals were registered with their respective breed registry organizations.

**Table 5:** Reason for death or euthanasia in hospital for 23 horses.

**Reason for death or euthanasia**	**Frequency (*n*, %)**
Progression of infection	6/23, 26.1%
Laminitis	3/23, 13.0%
Hoof wall separation	3/23, 13.0%
Gastrointestinal rupture; septic peritonitis	2/23, 8.7%
Fracture severity	2/23, 8.7%
Declining overall clinical condition	2/23, 8.7%
Sepsis	2/23, 8.7%
Hemorrhage	1/23, 4.3%
End-stage osteoarthritis	1/23, 4.3%
Cardiovascular arrest	1/23, 4.3%

## Discussion

The implementation of effective analgesic strategies for hospitalized equine patients is imperative to maximize case outcome and welfare. Epidural catheter placement for repeated neuraxial analgesia is performed clinically in our hospital as a multimodal strategy. The primary objective of this study was to report the response to therapy and to critically examine the complications observed in hospitalized horses undergoing epidural catheterization and treatment. Secondary objectives included description of the case population undergoing epidural catheter placement and therapy for long-term analgesic management and case outcome.

Epidural catheters were placed most frequently in cases with orthopedic pathologies, which is consistent with the results of a previous retrospective study (18). Synovial sepsis was the most common specific diagnosis prompting epidural catheter placement, which was also a common condition in the prior study (18). Epidural catheters and epidural analgesia are particularly well-suited for orthopedic conditions, particularly those involving the hind limbs (3, 14, 19, 31). The efficacy of epidural analgesia for septic arthritis, a particularly painful inflammatory condition, has also been previously documented (6, 9, 18, 32). Caudal epidural catheters and caudal epidural analgesia have been used for hindlimb as well and forelimb conditions (3, 14, 19, 33, 34). Cervical epidural catheter placement and analgesia has recently been described as an alternative neuraxial technique for thoracic limb conditions (35–37). It is important to consider, however, that caudal epidural analgesia can also be an effective strategy for non-orthopedic conditions including abdominal and urogenital conditions (18, 20, 25). The epidural catheters in this study were maintained for a median of 4 days, with a maximum duration of 33 days. Although an epidural catheter has been maintained in a mare for 56 days (21), others describe a maximum duration of 20–28 days (3, 18).

The initial response to epidural therapy with morphine was considered positive for the majority (59.7%) of the catheters. Morphine was used in every dose of epidural analgesic administered in this study. Morphine, a pure μ-opioid agonist, when administered epidurally is believed to diffuse across the dura mater and bind to opioid receptors in the spinal cord dorsal horn (3). Morphine has the advantage of lower lipid solubility relative to other opioids such as fentanyl, which prolongs the duration of action (38). The analgesic efficacy of epidural morphine has been well-documented in horses (3, 5, 9, 14, 21, 26, 27, 31, 34, 36, 38, 39). Adjunctive therapeutic agents, such as alpha-2 agonists or local anesthetics, were administered concurrently for some doses in this study. The synergistic effects of epidural administration of morphine and an alpha-2 agonist can provide enhanced and prolonged analgesia (3, 5, 7, 14, 17). Although epidural administration provides opioid analgesia locally at the level of the spinal cord, supraspinal, and systemic effects are also observed (3). These local and systemic effects can contribute to the development of adverse events.

Complications were observed more frequently in this study than what has been previously reported (18, 24), with complications reported for 74.2% of the epidural catheters. It is important to note, however, that the majority (51.6%) of complications were considered to be mild and did not require medical intervention. An all-inclusive definition of complication was used to meet our objective to report and critically evaluate the clinical considerations of epidural catheterization and dose administration in hospitalized equine patients. By documenting and understanding these observed complications, improvements for monitoring and complication management can be integrated to facilitate continued use of this effective analgesic strategy. Complications were categorized as technical, patient-related, and exaggerated physiologic responses (18).

Technical complications related to problems with the epidural catheter system have previously been reported for people and veterinary species (18, 28, 40, 41). In the retrospective study by Martin et al., technical complications were observed for 22 of 50 catheters and included catheter dislodgement, dislodgement of the catheter adapter or filter, obstruction of the catheter, and leaks from any part of the catheter system (18). Technical complications were observed in 12 of 62 epidural catheters in our study, however we used a modified definition of technical complication to separate issues into those due to a problem with the epidural catheter itself vs. those due to damage to the catheter system caused by the patient (18). The most frequent technical complication observed in our study was due to catheter obstruction. A loss of patency has been previously observed and is believed to be due to local inflammation and fibrosis (18, 24). Potential other sources of catheter obstruction include kinking of the catheter in the epidural space or at the insertion site or plugging with fibrin or a blood clot (40). A contribution of epidural hemorrhage to catheter occlusion has also been explored, although this effect was not observed in the horses in our study (24). Based on individual clinician preference, a variable volume of 0.9% saline or heparinized saline solution was “flushed” into the epidural catheter following analgesic dose administration. This practice was inconsistent within and between cases, which prevented thorough analysis. A closer investigation into this practice is necessary, as the volume of flush solution administered could influence the distribution of the analgesic administered and could also potentially influence the local inflammatory response (7, 42). Leaking at the catheter insertion site is another potential technical complication and has been observed in people and horses (18, 43). The improper connection of the elements of the epidural catheter system or at the entry site could be due to a technical error or due to self-trauma or damage.

Patient-related complications were categorized as events during which the patient caused damage to the catheter. These complications, such as self-removal, were observed in 14 of 62 catheters. Catheter dislodgement has been observed in people, horses, and dogs (18, 41, 43). Catheter dislodgement has been attributed to issues with fixation of the catheter at the skin, characterized by rigid skin fixation with loose catheter movement under the skin causing subsequent dislodgement (18, 44). Alternatively, retrograde flow of epidural injectate solutions due to reduced compliance of the epidural space can cause the catheter to migrate (18, 44). Seven situations of catheter dislodgement were observed in this study. The catheter was found removed or partially removed in these instances, and the removal was not witnessed. Although the above-described causes of dislodgement cannot be excluded, pruritus was concurrently observed for 4/7 catheters, and the contribution of pruritus leading to catheter removal cannot be excluded. Opportunities to prevent patient damage to the catheter, such as the use of a protective dressing and removal of hay racks or other stall elements that could traumatize the epidural catheter system are selected routinely in our hospital to minimize complications. The protective dressings were not standardized in this study, but they typically consisted of an adhesive dressing permitting protection of the site while still concurrently allowing site examination. Despite dislodgement or damage to the overlying protective bandage and/or catheter system, there were no instances of catheter breakage and retention. Monitoring of the epidural catheter by the clinical staff was important to identify and intervene in these technical and patient-related complications.

Exaggerated physiologic responses were observed most frequently of the complication categories. These were observed or potential behaviors or physiologic consequences of epidural analgesic administration. A contribution of skin irritation and inflammation from the adhesives and dressing used to secure the epidural catheter system should also be considered. Agitation was the most frequently observed physiologic response, and this frequency has not been previously recognized. As all epidural doses contained morphine, the agitation observed could have been related to the morphine administration. Systemic opioids are known to cause agitation and locomotor stimulation in horses (3, 13, 45). Epidural morphine administration and excitatory behaviors in horses has been described and is believed to be due to the effects at opioid receptors within the central nervous system (3, 7, 26). In an equine case report, a strong excitatory phase characterized by increased locomotor activity, dysphoria, and photophobia lasting several hours was observed (21). In the previous clinical retrospective study, two horses demonstrated muscle tremors, but no other excitatory behaviors were observed (18). Since the muscle tremors were not repeatable, the authors concluded that the tremors were unlikely caused by the morphine (18). However, tremors with opioid administration have been observed, and as with our study, intra- and inter-individual variation in behaviors was observed (13). Our study was reliant upon data collected from subjective patient assessments. Objective data regarding the onset and specifics of these agitated behaviors was not available and the contribution of pain itself to the agitation observed should also be considered. Future investigations will concentrate on further characterizing the etiology and management of these excitatory behaviors. Dopamine and opioid receptor antagonists have been investigated as a possible treatment for the opioid-induced adverse excitatory behaviors (3). Trazodone, a serotonin receptor antagonist and reuptake inhibitor, was administered to some of the horses in this study, and studies investigating the effects of trazodone on opioid-induced agitation and excitatory behaviors are warranted.

Pruritus was the second-most frequently observed exaggerated physiologic response seen in 29.0% of the epidural catheters. Pruritus is a common sequela of opioid neuraxial analgesia in people, with moderate or severe pruritus reported in 15.4% of patients reported in one study, and observations up to 30–100% have also been documented (46, 47). Pruritus has been documented in horses following epidural morphine administration in a limited number of case reports (48–50). Despite the frequency of pruritus observed in our case population, it was not observed in the prior retrospective clinical study by Martin et al. (18). In our study, the pruritus was not graded objectively, and reports were based on descriptions in the patient notes. The descriptions included verbiage describing a range in severity from mild to severe and continuous. Severe pruritus can lead to damage to the catheter system, skin irritation and cutaneous breakdown with potential subsequent infection, increased discomfort, and patient distress leading to premature cessation of the analgesic strategy (28, 46, 49). Neither histamine release nor the composition of the morphine solution is believed to cause the pruritus, and the exact mechanism is not well-understood (47, 49). The “itch center” within the central nervous system, activation of the medullary dorsal horn, antagonism of inhibitory neurotransmitters and pathways, and the role of prostaglandins have been investigated, with likely important individual variation (47). Multiple therapeutic options have been explored for people including opioid antagonists, propofol, NSAIDs, droperidol, and 5-HT_3_ antagonists (47, 51). Future studies specifically and objectively characterizing neuraxial-induced pruritus and the relationship to pain pathways are imperative for equine patients.

The gastrointestinal effects of opioids administered *via* systemic and epidural routes have been explored in many species including horses (13, 17, 39, 52, 53). However, these effects in hospitalized cases are clinically observed but less understood. A reduction in manure production was observed in the horses in this study, which was also observed in a horse previously undergoing long-term epidural treatment (21). The contribution of epidural opioid administration to decreased gastrointestinal transit, impaction, and rupture is likely multifactorial, and the horses in this study had multiple pre-disposing factors for gastrointestinal disturbances including pain, systemic drug administration, general anesthesia, procedural sedations, stall confinement, and diet change. Unfortunately, due to the medical record system, further objective detail regarding manure production and gastrointestinal motility could not be elucidated. In future studies with more intensive observation and monitoring, the direct contribution of epidural analgesia to the development of gastrointestinal complications can be explored. Careful gastrointestinal monitoring including physical examination parameters, manure output, and behavioral assessments should be performed and interventions such as enteral fluid administration and manual rectal evacuation may be necessary based on the information provided in this study.

Neurologic sequelae of epidural analgesia were observed with dose administration, and ataxia and recumbency have been reported in prior studies (3, 17, 18, 28). Morphine administered epidurally alone does not cause motor impairment (3, 7). However, if the dose is administered in a large volume or too rapidly, ataxia may be observed due to a local compressive effect (7, 28, 54, 55). This is particularly important during pregnancy and in situations of obesity, during which the anatomy of the epidural space is altered leading to alterations in the cranial spread of the epidural dose (28, 55). Although not observed in any of the horses in our study, seizures and upward fixation of the patella have also been observed following epidural administration (18, 56, 57). We were unable to correlate the observed instances of ataxia with the speed of dose administration. Slow administration of ~1 mL every 10 s has been recommended (28).

Urinary retention in horses with epidural analgesia has not been previously reported, to the authors' knowledge. However, urinary complications are well-described in people and dogs undergoing epidural treatment (41, 43, 46, 58). It is our clinical impression that urinary sequelae may occur more frequently than we observed. Due to the differences in management of equine patients vs. that of people and dogs, we believe urinary retention may go unrecognized without careful observation. The results of this study support careful monitoring of urinary signs with continuous epidural analgesia treatment, as intervention such as urinary catheterization may be required to prevent serious patient injury.

The cases included in this retrospective study were administered multiple medications concurrently during the time of epidural catheterization and treatment. The majority of cases received systemic antimicrobial therapy for the primary condition. No evidence of infection at the epidural catheter site was observed for any of the epidural catheters in this study. However, objective microbial analysis of the catheter site or the catheter was not performed. Pseudomonas aeruginosa was cultured from one epidural catheter entry site in the previous retrospective study, and aseptic placement of the catheter system, aseptic dose administration, and site monitoring is imperative to monitor for the potential catastrophic development of an epidural abscess (18, 28). Signs of sepsis and coagulopathy are traditionally considered contraindications for epidural catheterization and dose administration due to the risk of epidural infection and hematoma (2, 19). However, horses with evidence of sepsis and those that were administered clopidogrel for thromboprophylaxis did not have any documented infection or hemorrhage, respectively, at the epidural catheter site. It is important to note that caution should still be exercised for cases with signs of sepsis or coagulation disorders until the results of larger studies in horses are available.

Discharge from the hospital was achieved for 52.1% of the horses in this study. Epidural catheterization and analgesia are often elected for challenging hospitalized cases with multiple severe systemic pathophysiologic processes. This is reflected in the high frequency of observed complications during hospitalization not related to the epidural catheter (69.3%). A contribution of epidural analgesia could not be excluded for 4/23 of the horses that did not survive, and these cases had multiple confounding factors likely contributing to their death. Thus, death or euthanasia in hospital was more frequently due to the severity of the horses' underlying disease and was not related to epidural therapy. A limited number of pregnant mares were included in this study. Epidural analgesia has been used with subsequent successful delivery of a live foal in this study and in a prior case report (21). Studies specifically examining the effect of epidural analgesia and opioid receptor distribution on the gravid equine uterus are required.

This study was reliant upon the data available in our hospital's digital and paper medical records system. The results were dependent upon description of catheter placement and complications in the record. We did not have consistent data regarding the placement of the epidural catheter system, the number of attempts to place the catheter, and the exact specifications of the adhesive dressing. We did not use an objective system to evaluate discomfort behaviors, thus the response to epidural treatment or lack thereof was a subjective assessment. The frequency of patient monitoring was based on clinician preference and underlying disease and was not standardized. Thus, it is possible that behaviors or adverse events were under-reported if they were not directly observed. As the availability of remote monitoring techniques increases, the ability to document and investigate these complications will increase.

The frequency of complications reported in this study demands frequent patient monitoring. Although we separated observed complications into categories for the presentation of data and analysis, these complications can be interconnected. For instance, exaggerated physiologic responses, such as pruritus, can lead to patient-related and technical complications such as catheter system dislocation, if a patient intervention is not achieved in a timely manner. Refinements of technical strategies may lead to reduction of complication frequency with further experience in equine epidural catheter placement and dose administration. Epidural analgesia is an effective strategy for pain management, and the frequency of reported complications should not deter catheter placement if it is clinically indicated. However, the ability to monitor and address these complications described and identified in this study should be considered if epidural catheter placement is elected.

## Data availability statement

The raw data supporting the conclusions of this article will be made available by the authors, without undue reservation.

## Ethics statement

Ethical review and approval was not required for the animal study because of the observational nature of the study. Written informed consent was obtained from the owners for the participation of their animals in this study.

## Author contributions

HD and KH contributed to the design, organization, and execution of this study. HD, DF, KH, and BD contributed case information. HD and KS performed the case review and data entry. HD and MM performed the data analysis. HD composed the manuscript. All authors revised and endorsed the final manuscript.

## Conflict of interest

The authors declare that the research was conducted in the absence of any commercial or financial relationships that could be construed as a potential conflict of interest.

## Publisher's note

All claims expressed in this article are solely those of the authors and do not necessarily represent those of their affiliated organizations, or those of the publisher, the editors and the reviewers. Any product that may be evaluated in this article, or claim that may be made by its manufacturer, is not guaranteed or endorsed by the publisher.
